# Daptomycin-Biomineralized Silver Nanoparticles for Enhanced Photothermal Therapy with Anti-Tumor Effect

**DOI:** 10.3390/polym14142787

**Published:** 2022-07-07

**Authors:** Jie Zhang, Jing Wang, Guixiu Fan, Bingjie Zhang, Guanglong Ma, Haiyan Xiao, Longgang Wang

**Affiliations:** 1 Key Laboratory of Applied Chemistry, Nano-Biotechnology Key Lab of Hebei Province, Hebei Key Laboratory of Heavy Metal Deep-Remediation in Water and Resource Reuse, College of Environmental and Chemical Engineering, Yanshan University, Qinhuangdao 066004, China; 19991641458@163.com (J.Z.); wjpersonal@yeah.net (J.W.); 17854253633@163.com (G.F.); wingsbingjie@163.com (B.Z.); 2 State Key Laboratory of Metastable Materials Science and Technology, Yanshan University, Qinhuangdao 066004, China; 3 Centre for Cancer Immunology, Faculty of Medicine, University of Southampton, Southampton SO166YD, UK; g.ma@soton.ac.uk

**Keywords:** daptomycin, silver nanoparticle, biomineralization, anti-tumor, biocompatibility

## Abstract

Silver nanoparticles as photothermal agents have the problems of low stability and low photothermal conversion efficiency. Amphiphilic daptomycin can improve the stability of silver nanoparticles, thereby improving their photothermal conversion efficiency. Herein, daptomycin-biomineralized silver nanoparticles (Dap-AgNPs) were prepared by reducing silver nitrate with sodium borohydride in the presence of daptomycin as a stabilizer and biomineralizer. The Dap-AgNPs had good solution stability and peroxidase-like activity. Furthermore, the photothermal conversion efficiency of the Dap-AgNPs was as high as 36.8%. The Dap-AgNPs displayed good photothermal stability under irradiation. More importantly, the Dap-AgNPs showed good cell compatibility with HeLa cells and HT-29 cells without irradiation by 808-nanometer near-infrared light at a concentration of 0.5 mM, and the cell viability was greater than 85.0%. However, the Dap-AgNPs displayed significant anti-tumor ability with irradiation by 808-nanometer near-infrared light, which was due to the increasing temperature of the culture medium caused by the Dap-AgNPs. In conclusion, Dap-AgNPs have potential applications as photothermal agents in the treatment of tumors.

## 1. Introduction

In the past few decades, chemotherapy, radiotherapy, and surgery have been the conventional treatments for cancer in clinical practice [1,2]. Compared with conventional tumor treatment methods, photothermal therapy (PTT) has many advantages, such as its minimally invasive character, low side effects, and high specificity [3,4]. Therefore, PTT has attracted increasing attention due to its unique advantages [5]. PTT has a small high-temperature hot-spot generated by direct contact between the tumor area and photothermal agents [6]. The thermal effect generated by light is concentrated on the target, thereby achieving targeted therapy and reducing damage to normal tissues [7]. Various nanomaterials, including MoS_2_ [8], graphene [9], ICG-loaded nanoparticles [10], and precious-metal nanoparticles [11], have been widely used as photothermal agents. The nanoparticles of precious metals, such as gold, silver, and palladium stand out in PPT due to their unique properties [12]. The type, size, shape, and stability of precious-metal nanoparticles affect their photothermal properties [12,13]. However, it is still necessary to improve the photothermal properties of precious-metal nanoparticles.

Silver nanoparticles (AgNPs) have photothermal effects, which are mainly due to their localized surface plasmon resonance (LSPR) capability [14]. The LSPR property of silver nanoparticles is correlated with their morphologies, sizes, and solution stability [15]. The current methods for synthesizing silver nanoparticles mainly use reducing agents, such as sodium borohydride and sodium citrate, to reduce the silver nitrate solution [16]. However, silver nanoparticles prepared only under the action of reducing agents usually have problems such as poor stability and low photothermal conversion efficiency, which greatly limits their application in the field of biomedicine [15]. The preparation of novel silver nanoparticle photothermal agents with good stability and high photothermal conversion efficiency is more conducive to the research on PTT [17]. Chen et al. prepared bimetallic Au-Ag nanoparticles with high absorbance intensity in the visible region by adjusting the molar ratio of Ag to Au precursors. The photothermal conversion performance of the bimetallic Au-Ag nanoparticles was 31.41% [18]. Taehoon Park et al. synthesized highly PEGylated bovine serum albumin (BSA)-coated silver nanoparticles loading ICG (PEG-BSA-AgNPs/ICG). The accumulating of the nanoparticles in tumors enabled the tumor surface temperature to be raised to 50 °C by laser irradiation and successfully inhibit tumor growth [19]. Dasom Kim et al. synthesize bovine serum albumin (BSA)-coated silver nanoparticles (BSA-AgNPs), which exhibited considerable photothermal conversion ability and significant cytocidal effects on B16F10 melanoma cells [20]. Bian et al. used octreotide as a template to synthesize stable silver nanocages (AgNCs) by adjusting the incubation time and AgNO_3_ dosage to control the growth-kinetic rate of AgNCs. The prepared AgNCs had a high photothermal conversion efficiency and could effectively ablate tumors under near-infrared (NIR) light irradiation [21]. Tong et al. used reduced graphene oxide (rGO) to simultaneously anchor AgNPs and daptomycin to prepare rGO@Ag@Dap nanocomposites. The rGO@Ag@Dap nanocomposites had a significant antibacterial ability [22].

The daptomycin (Dap) used in this study, which has a molecular weight of 1621, is an amphiphilic antimicrobial peptide. Each Dap molecule has three primary amino groups, four carboxyl groups, and one hydroxyl group. These groups give Dap a strong hydrophilic property. Dap also has many hydrophobic groups [23]. Amphiphilic Dap self-assembles into micelles that act as stabilizing agents and soft templates. Thus, the Dap micelles were used to prepare stable daptomycin-biomineralized silver nanoparticles (Dap-AgNPs). The Dap micelles interacted with silver ions and underwent biomineralization under the action of a strong reducing agent, sodium borohydride, resulting in the formation of stable Dap-AgNPs. We performed an elemental characterization of the Dap-AgNPs, tested their photothermal properties, stability, and cytotoxicity, and finally evaluated the photothermal effect of Dap-AgNPs on tumor cells. This is the first report on the use of Dap as a template for the synthesis of silver nanoparticles.

## 2. Materials and Methods

### 2.1. Materials

Daptomycin was purchased from Yuancheng Gongchuang Technology Co., Ltd. (Wuhan, Hubei, China). Acetic acid, sodium borohydride (NaBH_4_, 98%), silver nitrate (AgNO_3_, 98%), and thiazole blue (MTT, 98%) were purchased from Aladdin (Shanghai, China). Fluorescein diacetate (FDA, 95%) and propidium iodide (PI) were purchased from Beyotime Biotechnology Co., Ltd. (Beijing, China). Fetal bovine serum was purchased from Tianhang Biotechnology Co., Ltd. (Hangzhou, Zhejiang China). Pancreatin was purchased from Xiangbo Biotechnology Co., Ltd. (Guangzhou, Guangdong, China). The reagents used in the experiments were all analytical reagents. HeLa cells were purchased from Center for Typical Culture Collection (Wuhan, Hubei, China).

### 2.2. Preparation of Dap-AgNPs

In total, 10 μL of acetic acid solution (0.2 M), 50 μL of Dap solution (4 mM), 50 μL of AgNO_3_ solution (25 mM), and 100 μL of deionized water were sequentially added to a 2-milliliter polyethylene (PE) tube. After 1 min, 50 μL of freshly prepared NaBH_4_ solution (50 mM) was added. Ten minutes later, deionized water was added to obtain 500 μL of solution. The final solutions were dialyzed against deionized water using dialysis bag with the molecular weight cut off (MWCO) for 1000 to obtain Dap-AgNP solution.

### 2.3. Preparation of AgNPs

In total, 10 μL of 0.2 M acetic acid solution, 50 μL of deionized water, 50 μL of 25 mM AgNO_3_, and 100 μL of deionized water were added to a 2-milliliter PE tube. After one min, 50 μL of freshly prepared 50-millimolar NaBH_4_ solution was added. Ten minutes later, the solution was added to deionized water and diluted to obtain 1 mL of 1.25 mM AgNPs solution.

### 2.4. Determination of Peroxidase-like Property

The determination of peroxidase-like property was performed according to a previous report [24]. In total, 900 μL of 3,3′,5,5′-tetramethylbenzidine (TMB) (0.6 mM), 100 μL of H_2_O_2_ (0.3 M), 200 μL of HAc-NaAc (pH = 4.0, 0.2 M), and 200 μL of Dap-AgNPs (C_Ag_ = 0.83 mM) were mixed in a 2-milliliter PE tube at 25 °C for 10 min. A UV-Vis spectrometer was used to record the spectra changes of the reaction solution in the wavelength range of 500–800 nm. The control experiment was carried out under the same conditions.

### 2.5. Photothermal Performance of Dap-AgNPs

To evaluate the photothermal conversion effect of the Dap-AgNPs, 1 mL of Dap-AgNPs solution was placed in a PE tube and irradiated with an 808-nanometer NIR laser at 1.75 W/cm^2^ for 10 min. Dap solution, AgNP solution, and deionized water were also measured. After 10 min, the Dap-AgNP solution was naturally cooled to room temperature, and the laser was switched on and off 5 times to detect the photothermal properties of Dap-AgNPs. The photothermal conversion efficiency of Dap-AgNPs was calculated by irradiating with an 808-nanometer laser with a power of 1.75 W/cm^2^ until the temperature no longer changed, after which the laser was turned off and the solution was allowed to cool naturally. The photothermal conversion efficiency (*η*) of Dap-AgNPs was calculated from Formula (1) [25], as follows:

(1) $$\eta =\frac{hS(T_{max}-T_{sur})-Q_{Dis}}{I(1-10^{-A_{\lambda 808}})}$$

where *h* refers to thermal conductivity; *S* refers to the surface area of the container; *T_max_* refers to the maximum temperature; *T_sur_* refers to the ambient temperature; *Q_Dis_* refers to the light energy absorbed by the container; *I* refers to the light-source power; *A_λ_*_808_ refers to absorption of near-infrared light at 808 nm.

### 2.6. Cytotoxicity of Dap-AgNPs

HeLa cells, as human cervical cancer cells, and HT-29 cells, as human colon cancer cells, were used as typical tumor cells in the MTT assay [25]. HeLa cells and HT-29 cells were seeded separately in 96-well plates, and 200 μL of culture medium was added and incubated at 37 °C for 24 h. The existing medium was then replaced with 200 μL of the sample solution in DMEM medium. Cells were incubated at 37 °C for 24 h. In total, 100 µL of 0.5 mg/mL MTT replaced the medium containing the sample. After 4 h, the MTT solution was aspirated, and 150 μL of dimethyl sulfoxide was added to each well. After 10 min, the absorbance of each well was measured at 490 nm with a multi-plate reader. Cell viability was calculated according to Equation (2).

(2) $$\mathrm{Cell}\;\mathrm{viability}\;(\%)=\frac{OD_{sample}}{OD_{control}}\times 100$$

### 2.7. Anti-Tumor Effect In Vitro

HeLa cells in logarithmic growth phase were seeded into 96-well plates and cultured in a constant temperature incubator for one day. Next, the medium in each well was replaced with 200 μL of sterile sample solutions (Dap, AgNPs, and Dap-AgNPs) and incubated for 4 h, respectively. Subsequently, cells were irradiated with 808-nanometer laser at a power of 1.75 W/cm^2^ for 10 min. After the irradiation was completed, the cells were placed in an incubator for 12 h. Subsequently, cells were stained by FDA and PI and photographed.

## 3. Results and Discussion

### 3.1. Preparation of Dap-AgNPs

**Scheme 1:**
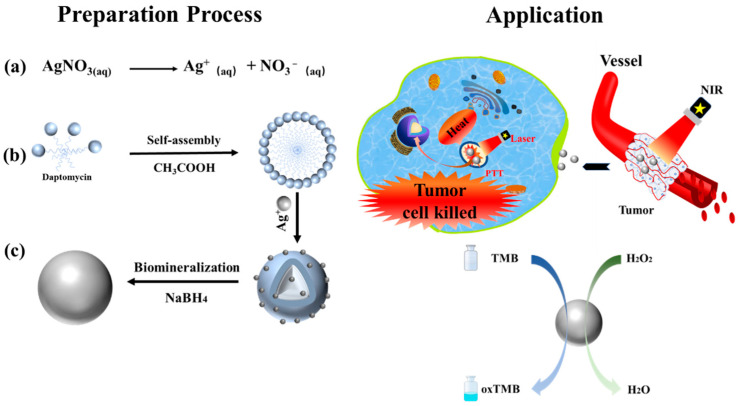
The preparation and application of stable daptomycin-biomineralized silver nanoparticles (Dap-AgNPs).

In this study, the Dap micelles and silver ions were complexed, and they were added with NaBH_4_ to form Dap-AgNPs with a specific structure through biomineralization. The silver precursor (AgNO_3_) was dissociated into a silver cation (Ag^+^) and a nitrate anion (NO_3_^−^) (Scheme 1, Step a) [26]. The Dap molecules self-assembled to form Dap micelles, with a large number of amino groups and carboxyl groups on the surface. Therefore, the Ag^+^ should have chemical complexation with the Dap (Scheme 1, Step b). Next, the Dap-AgNPs with a regular square structure were prepared through the effect of biomineralization (Scheme 1, Step c).

### 3.2. Characterization of Dap-AgNPs

**Figure 1:**
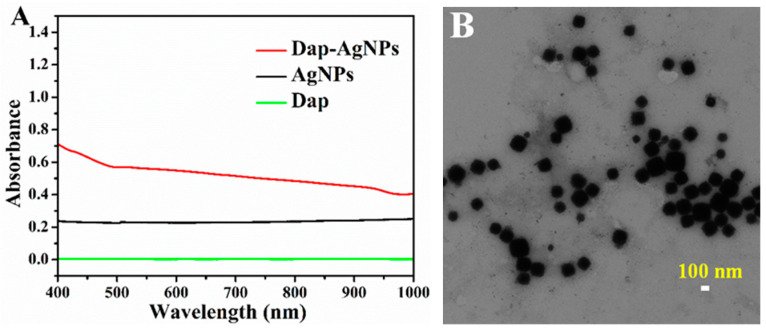
(**A**) The UV-Vis absorption spectra of sample solution; (**B**) transmission electron micrograph of Dap-AgNPs.

According to the formula of the photothermal conversion efficiency, we intuitively observed that the photothermal performance of the nanoparticles was closely related to the UV-Vis absorbance of the solution. As shown in Figure 1A, the Dap, as an amphiphilic antimicrobial peptide, had almost no absorbance in the range of 400–1000 nm. Compared with the AgNPs, the Dap-AgNPs had a higher absorbance, which meant that the Dap-AgNPs had a better photothermal effect. The absorbance of the Dap-AgNPs (0.48) was much higher than that of the AgNPs (0.22), at 808 nm. This showed that the Dap had a great influence on the preparation process of the Dap-AgNPs. The groups of Dap interacted with the Ag^+^ in the solution. The Dap-AgNPs were formed under the guidance of the Dap after adding a reducing agent, NaBH_4_. The Dap-AgNPs we prepared were not simply a mixture of Dap and AgNPs. The Dap-AgNPs had good dispersibility and were stored stably for about one month at 4 °C. However, the AgNPs that were directly reduced with the NaBH_4_ without the Dap showed severe coagulation and preparation within 24 h, the supernatant was almost clear, and the coagulated nanoparticles were seen at the bottom. Therefore, the Dap had an important role as a stabilizer and template in the formation of the Dap-AgNPs. We tested the TEM after multiple dialysis. It can be seen from Figure 1B that the Dap-AgNPs presented a dispersed structure and about 138 ± 3.5 nm. In short, the prepared Dap-AgNPs had good absorbance, dispersibility, and stability in the solution.

**Figure 2:**
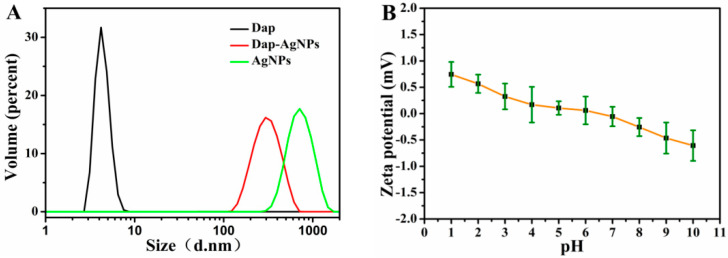
(**A**) Hydrodynamic size of each sample in solution and (**B**) zeta potential of Dap-AgNPs in different pH solutions.

To further analyze the state of the Dap-AgNPs in the solution, the hydrodynamic size and zeta potential of the Dap-AgNPs were measured [26]. As shown in Figure 2A, the hydrodynamic sizes of the Dap-AgNPs were about 221 nm, while the Dap self-assembled into micelles, whose hydrodynamic size was 4.2 nm, in solution. By contrast, the AgNPs were very unstable in solution and prone to coagulation; their hydrodynamic size was as large as 656 nm. The above results indicated that Dap can influence the formation and growth of silver nanoparticles through a certain force during the formation of silver nanoparticles, and the Dap-AgNPs that eventually formed were stably distributed in the solution. In summary, we successfully prepared Dap-AgNPs stabilized by Dap, and determined that the Dap probably guided the nucleation and crystal growth during the preparation process. The zeta potential of the Dap-AgNPs was around 0 mV under different pH, as shown in Figure 2B. The Dap probably contributed to slight changes in the zeta potential of Dap-AgNPs at different pH.

**Figure 3:**
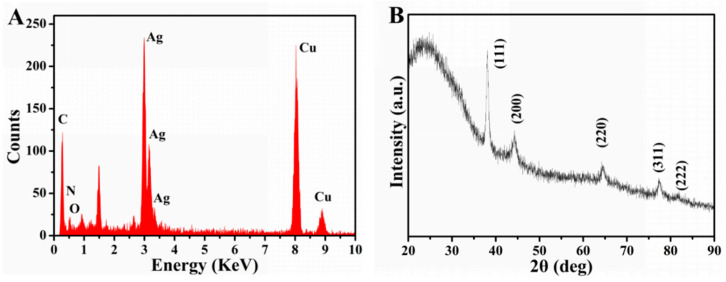
(**A**) EDS pattern and (**B**) XRD pattern of Dap-AgNPs.

According to the energy-dispersive spectroscopy (EDS) diagram in Figure 3A, the Dap-AgNPs contained N and O elements, which were derived from the amide bond and amino acid residues in the Dap, and the strong silver absorption peak was mainly derived from the silver nanoparticles. In addition, we performed an XRD characterization of the Dap-AgNPs. The results are shown in Figure 3B. The different diffraction peaks of the Dap-AgNPs were 38.15°, 44.27°, 64.58°, 77.06°, and 81.54° in 2θ, corresponding to (111), (200), (220), (311), and (222) crystal planes of the face-centered cubic structure of the silver.

### 3.3. Peroxidase-like Property of Dap-AgNPs

**Figure 4:**
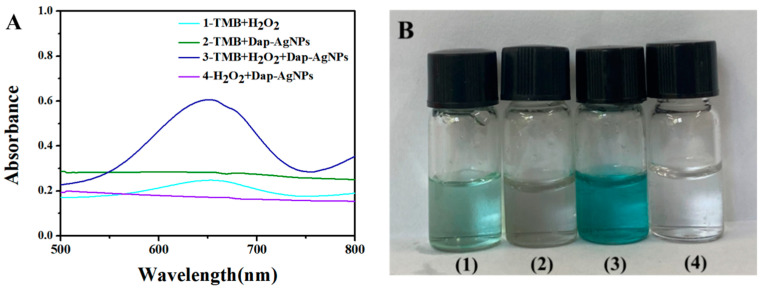
(**A**) UV-Vis absorption spectra of (**1**) TMB + H_2_O_2_; (**2**) TMB + Dap-AgNPs; (**3**) TMB + H_2_O_2_+ Dap-AgNPs; (**4**) H_2_O_2_ + Dap-AgNPs; (**B**) photographs of (**A**).

Dap-AgNPs may have peroxidase-like properties. Herein, colorless TMB was used as the substrate. The blue-color reaction of the TMB had an obvious absorption peak at 652 nm, indicating that oxidized TMB (oxTMB) was formed in this process. The high absorbance at 652 nm corresponded to an increase in TMB oxidation. As shown in Figure 4A, the absorbance of the (3) TMB + Dap-AgNPs + H_2_O_2_ group was much higher than that of the other controls, which indicated that the Dap-AgNPs effectively catalyzed the oxidation of the TMB. The absorbances at 652 nm of (1) TMB + H_2_O_2_, (2) TMB + Dap-AgNPs, (3) TMB + H_2_O_2_ + Dap-AgNPs, and (4) H_2_O_2_ + Dap-AgNPs at 652 nm were 0.248, 0.282, 0.605, and 0.171, respectively. Thus, (3) TMB + H_2_O_2_+ Dap-AgNPs had the highest absorbance values, which indicated the peroxidase-like activity of the Dap-AgNPs. In addition, the typical blue-product oxTMB appeared in the corresponding reaction solution, as shown in Figure 4B. This color phenomenon was similar to the antibacterial agent based on AgNPs and Fe_3_O_4_-loaded chitin microspheres previously reported by Yu [27]. Under acidic conditions, H_2_O_2_ may be broken down to hydroxyl radicals on the surface of AgNPs inside of Dap-AgNPs, and hydroxyl radicals caused the color of the solution to change from colorless TMB to blue oxTMB [28]. Dap enhanced the stability of the Dap-AgNPs in solution and made them catalytically active.

### 3.4. Photothermal Performance of Dap-AgNPs

**Figure 5:**
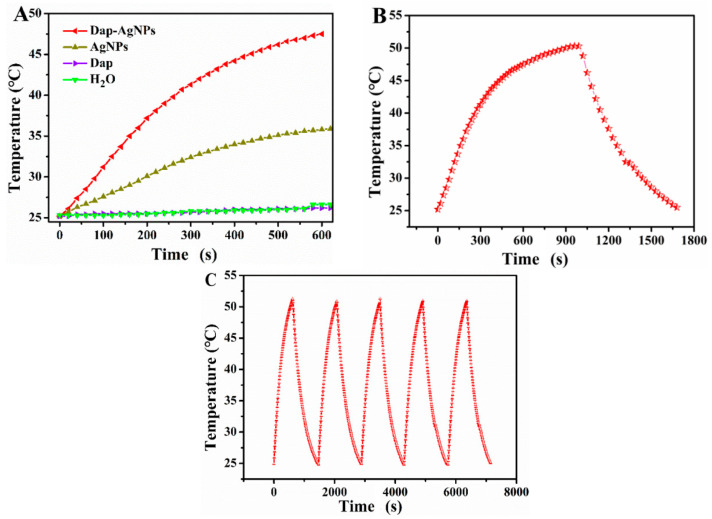
(**A**) The temperature of the sample solution irradiated with the 808-nanometer laser at 1.75 W/cm^2^ for 10 min; (**B**) the Dap-AgNPs solution irradiated with the 808-nanometer laser at 1.75 W/cm^2^ to reach maximum temperature and then cool down naturally; (**C**) temperature profile of Dap-AgNP solution during five cycles of laser irradiation.

Since silver nanoparticles must have a good temperature-increase effect when they are used for the thermal ablation of tumors, their photothermal conversion efficiency and photothermal stability are very important. The temperature changes of each group under 808 nm of NIR light with a power of 1.75 W/cm^2^ are shown in Figure 5A. The Dap-AgNPs solution reached 43 °C in 7 min, which was enough to cause a certain degree of damage to the cancer cells. The temperature of the solution was as high as 47 °C after 10 min of irradiation. However, the temperature of the AgNP solution was only 34 °C under the same continuous laser irradiation, while the temperature of the Dap solution and deionized water hardly changed under the same laser irradiation. To calculate the photothermal conversion efficiency of the Dap-AgNPs, we continued to irradiate until the temperature no longer changed; we then turned off the laser, and the Dap-AgNP solution was naturally cooled. The temperature changes over time are shown in Figure 5B. The photothermal conversion efficiency of the Dap-AgNPs was 36.8% according to the calculation. These results indicated that the prepared Dap-AgNPs had good photothermal conversion efficiency and increasing temperature effect.

We also tested the photothermal stability of Dap-AgNPs through continuous heating and cooling cycle. As shown in Figure 5C, in the case of five consecutive cycles of irradiation, the temperature of the Dap-AgNPs solution after irradiation was still able to reach 52 °C, which indicated that the Dap-AgNPs had good photothermal stability under laser irradiation. The above results revealed that the Dap-AgNPs prepared by us had good photothermal conversion efficiency and good photothermal conversion stability. This is because the use of the Dap as a template had a guiding effect on the growth and stability of the silver nanoparticles.

### 3.5. Cytotoxicity of Dap-AgNPs

**Figure 6:**
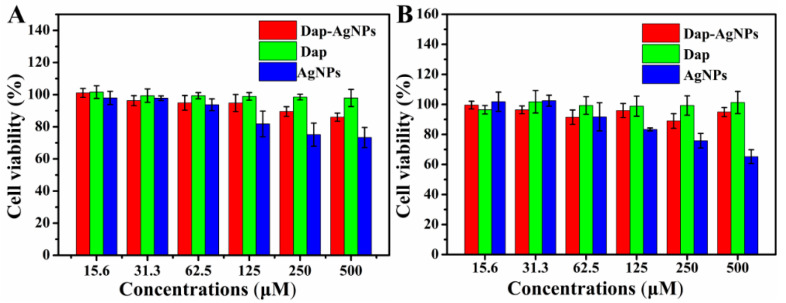
The biocompatibility of the sample to (**A**) HeLa cells and (**B**) HT-29 cells.

The cytotoxicity study was performed without 808-nanometer near-infrared light irradiation to determine the biocompatibility of the Dap-AgNPs. We used HeLa cells and HT-29 cells to test the biocompatibility of the Dap-AgNPs. As shown in Figure 6, the cell viability of the HeLa cells and the HT-29 cells of the Dap at various concentrations was greater than 90%. However, when the concentration of the AgNPs group was 0.5 mM, the cell viability of the HeLa cells and the HT-29 cells was 78.6% and 68.6%, respectively, which showed that the AgNPs had cytotoxicity. The reactive oxygen species generated by the oxidative stress may have been caused by the AgNPs, resulting in the loss of cell activity [29]. By contrast, even at a concentration of 0.5 mM, the cell viability of the Dap-AgNPs was more than 85.0%, indicating that the Dap-AgNPs had good biocompatibility, which greatly improved the medical prospects of the silver nanoparticles.

### 3.6. Anti-Tumor Performance of Dap-AgNPs

**Figure 7:**
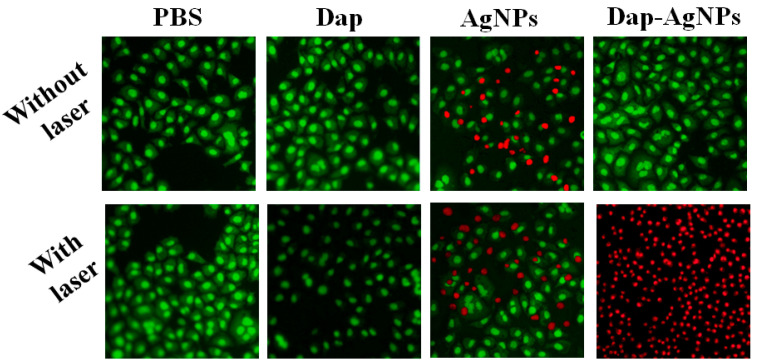
Pictures of HeLa cell stained by FDA (100 μg/mL) and PI (60 μg/mL) to observe the effects of each sample on the cells.

Using light to raise the temperature to kill cancer cells has become an effective anti-tumor method, and the Dap-AgNPs prepared by us have good photothermal conversion efficiency. We therefore observed the anti-tumor effect of Dap-AgNPs by applying laser irradiation. Similarly, we used HeLa cells in the experiment, as shown in Figure 7. The live cells had a green color, while the dead cells appeared red. The cells in the Dap-AgNPs group without laser treatment did not die. The Dap-AgNPs had a good inhibitory effect on the tumor cells under laser irradiation. In the phosphate-buffered saline (PBS) group and the Dap group, the cells treated with or without the laser remained live. The results showed that the NIR light did not harm the cells at a power of 1.75 W/cm^2^, and the Dap had no anti-tumor effect. As for the AgNPs prepared by the direct reduction in NaBH_4_ without the Dap, there were partial deaths of the cells with or without laser treatment. These results are essentially in line with the previous MTT results. In general, the Dap had good biocompatibility, and there was no anti-tumor effect under laser irradiation. However, the Dap-AgNPs had excellent anti-tumor effects under laser irradiation, which was probably due to the increasing temperature and release of Ag^+^ [30].

## 4. Conclusions

In conclusion, new Dap-biomineralized silver nanoparticles, Dap-AgNPs, were successfully prepared. The Dap-AgNPs had enhanced stability and photothermal properties compared with the AgNPs prepared without the Dap. The Dap-AgNP solution can be stored without precipitation at 4 °C for about 30 days, and the Dap-AgNPs’ photothermal conversion efficiency was as high as 36.8%. Notably, the Dap-AgNPs showed good biocompatibility with the HeLa cells and the HT-29 cells, and their cell activity remained above 85%. After 808-nanometer NIR light treatment, the Dap-AgNPs displayed good anti-tumor effects on the HeLa cells. The method of preparation of the Dap-AgNPs offers good potential for the design of drugs for anti-tumor applications.

## Data Availability

The data presented in this study are available on request from the corresponding author.
